# Supplementary figures and images for: Rhinovirus C replication is associated with the endoplasmic reticulum and triggers cytopathic effects in an in vitro model of human airway epithelium
Source: PLoS Pathog. 2022 Jan 7;18(1):e1010159. doi: 10.1371/journal.ppat.1010159 (PMC8741012; doi:10.1371/journal.ppat.1010159)

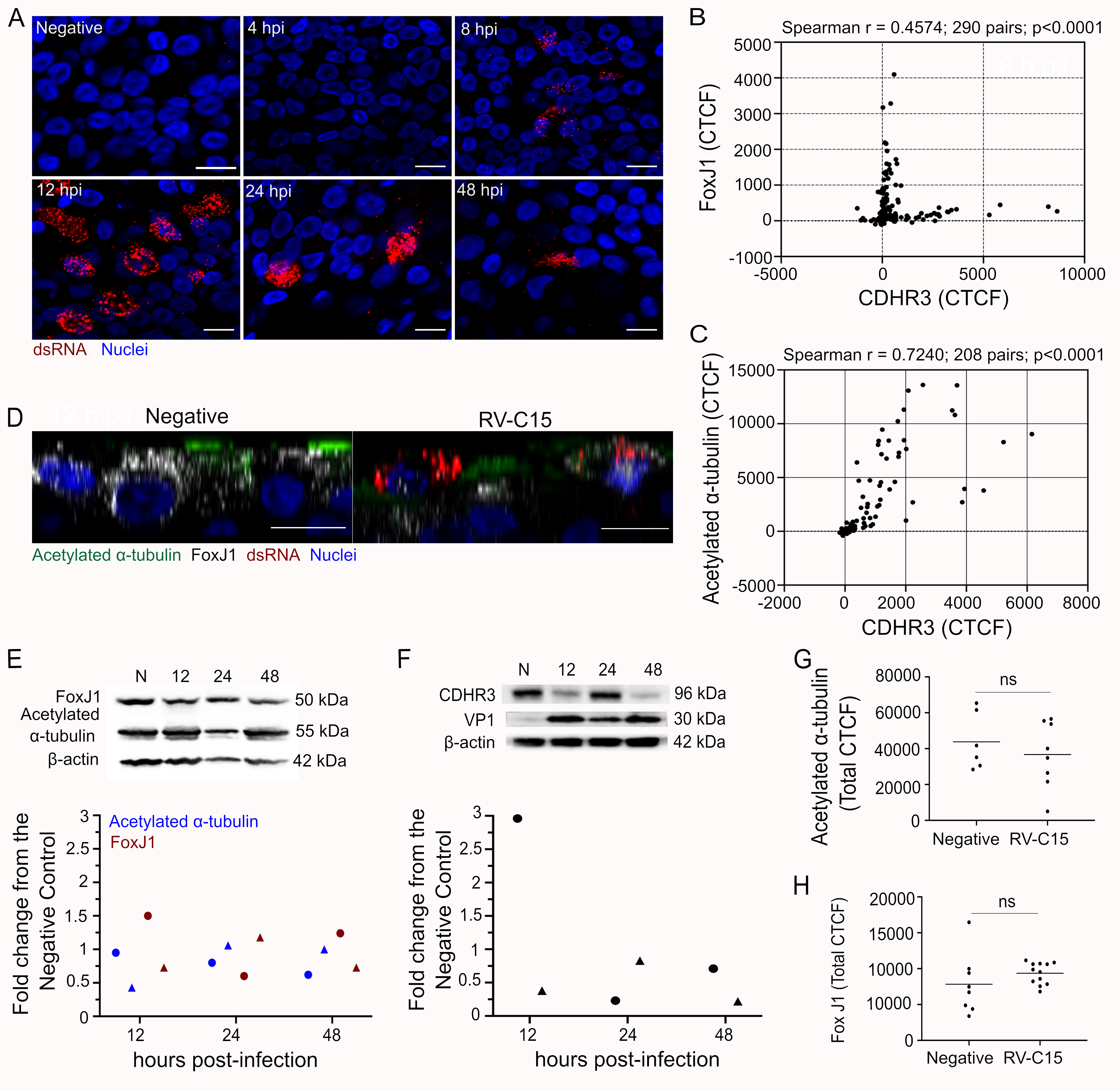

Supplement: S1 Fig — A: Immunofluorescence to detect dsRNA (red) in RV-C15 (1010 RNA)- infected HAE (nuclei, blue; scale bar = 10μm). B-C: Spearman correlation analysis (two-tailed; 0.95% confidence interval) between fluorescence levels of CDHR3 and FoxJ1 (B) or acetylated α-tubulin (C) in non-infected HAE at 12hpi. Each dot represents the fluorescence levels of CDHR3 and FoxJ1 or acetylated α-tubulin quantified in the same Z-slice. D: Orthogonal XY view from non-infected and RV-C15-infected (dsRNA+, red) HAE (nuclei, blue) stained by immunofluorescence for FoxJ1 (gray) and acetylated α-tubulin (green) at 12hpi (z-stacks at 1μm of thickness; scale bar = 10μm). E and F: Fold change graph represents FoxJ1 (E), acetylated α-tubulin (E), and CDHR3 (F) protein levels normalized to the endogenous control (actin) and compared to non-infected cultures. Data shown are from two independent donors, represented by circles and triangles. Blot above is from the donor represented by circles (E) and triangles (F); VP1 protein of RV-C15 was detected in cultures from the donor "triangle" used for both assays (E, F). G-H: Quantification of fluorescence levels (CTCF) for acetylated α-tubulin (G and FoxJ1 (H) in non-infected and RV-C15-infected (dsRNA+) HAE at 12hpi. Dots represent the total CTCF per cell and the line represents the mean. Statistical analysis was done using Mann-Whitney U test (Two-tailed; 0.95% confidence interval). (TIF) [file ppat.1010159.s001.tif]

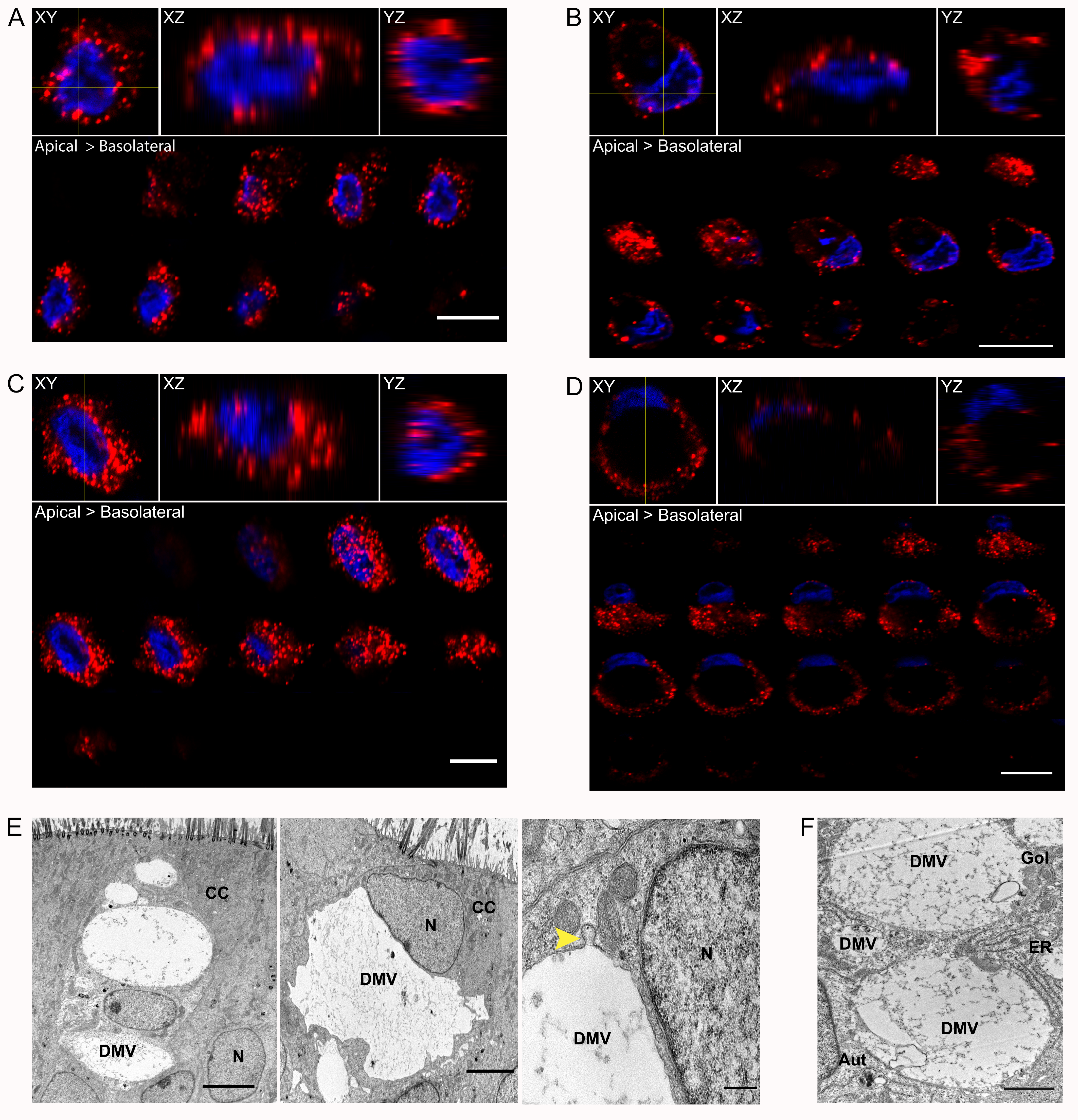

Supplement: S2 Fig — A-D: Orthogonal views (XY, XZ and YZ planes; yellow lines show the location of XZ and YZ views on the XY plane) from the RV-A16 (A-B) and RV-A2 (C-D) -infected HAE immunostained for dsRNA (red; nuclei, blue) at 12hpi (z-stacks at 1μm of thickness). Z-stacks at 1μm of thickness shows two profiles for dsRNA (red) detection by immunofluorescence at perinuclear (A, C) or close to the plasma membrane in a ring-like disposition (B, D) at 12hpi (scale bar = 10μm). E-F: Transmission electron microscopy of HAE infected with RV-A16 (E; scale bars = 5, 2, and 2μm respectively) or RV-A2 (F; scale bar = 2μm) at 12hpi. Visualization of ciliated cells (CC) with large, double-membrane vesicles (DMV; E and F) located above (E–left panel) or below the nucleus (E–middle panel); and the fusion of small vesicles to a larger vesicle (E–right panel; yellow arrow). N = nucleus; Gol = golgi; ER = endoplasmic reticulum; Aut = autophagosome. (TIF) [file ppat.1010159.s002.tif]

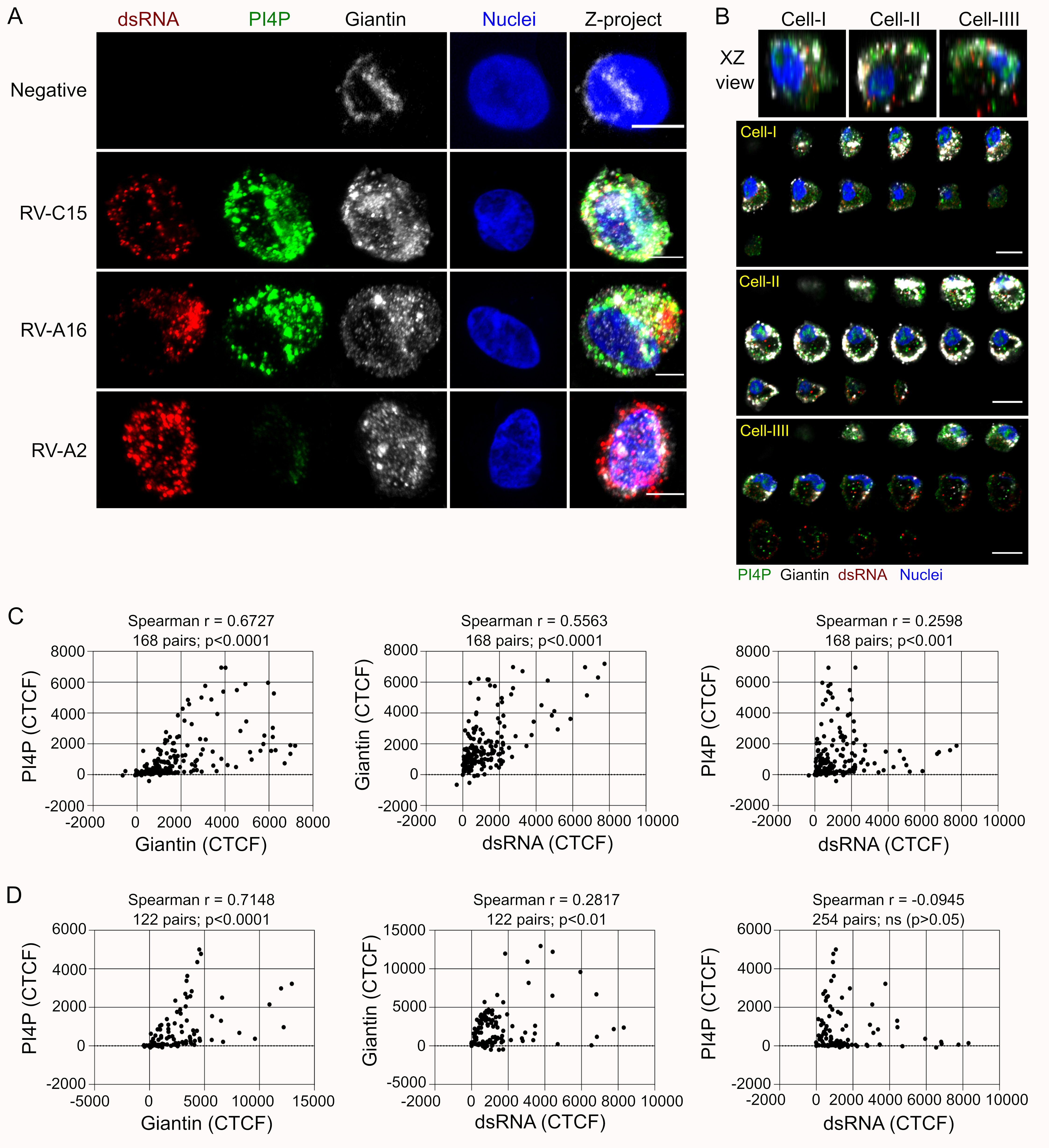

Supplement: S3 Fig — Maximum Z-projection for dsRNA (red), PI4P (green), and Giantin (gray) detection by immunofluorescence in non-infected and RV-infected HAE (scale bar = 5μm) (A). B: XZ orthogonal views (individual and montage of z-series) showing the differences in giantin (gray) and PI4P (green) distribution in RV-C15-infected cells with dsRNA (red) with either a perinuclear (cell I) or ring-like profile (cells II and III) (scale bar = 5μm). C-D: Spearman correlation analysis (two-tailed; 0.95% confidence interval) between giantin and PI4Pfluorescentlevels (CTCF) in RV-A16 (C) or RV-A2 (D) -infected HAE at 12hpi. Each dot represents the fluorescence levels of giantin and PI4P quantified in the same Z-slice. (TIF) [file ppat.1010159.s003.tif]

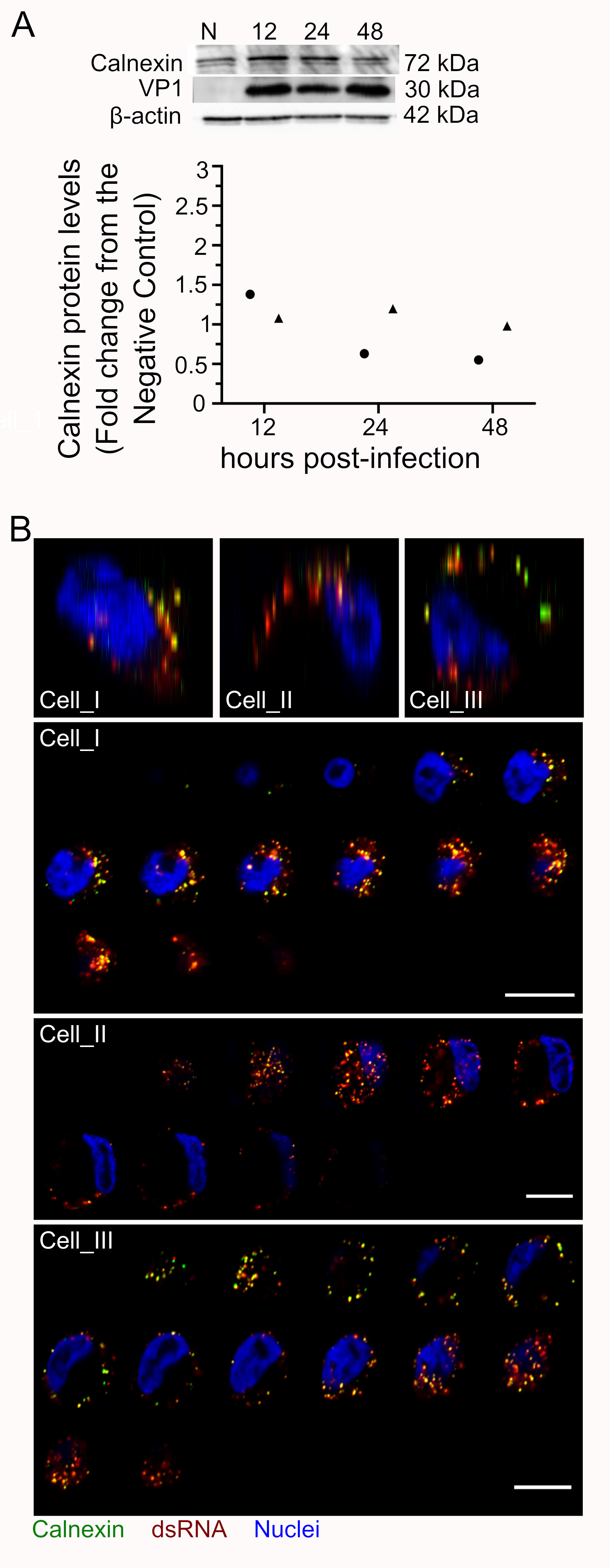

Supplement: S4 Fig — A: Fold change graph represents calnexin protein levels normalized to the endogenous control (actin) and compared to non-infected cultures. Data shown are from two independent donors, represented by circles and triangles. Blot above is from the donor represented by triangles, which includes the detection of VP1 of RV-C15. B: Orthogonal XZ views show the perinuclear (cell-I) and ring-like (cell-II and cell-III) pattern of dsRNA (red) and calnexin (green) in RV-C15 infected HAE (scale bar = 5μm). (TIF) [file ppat.1010159.s004.tif]

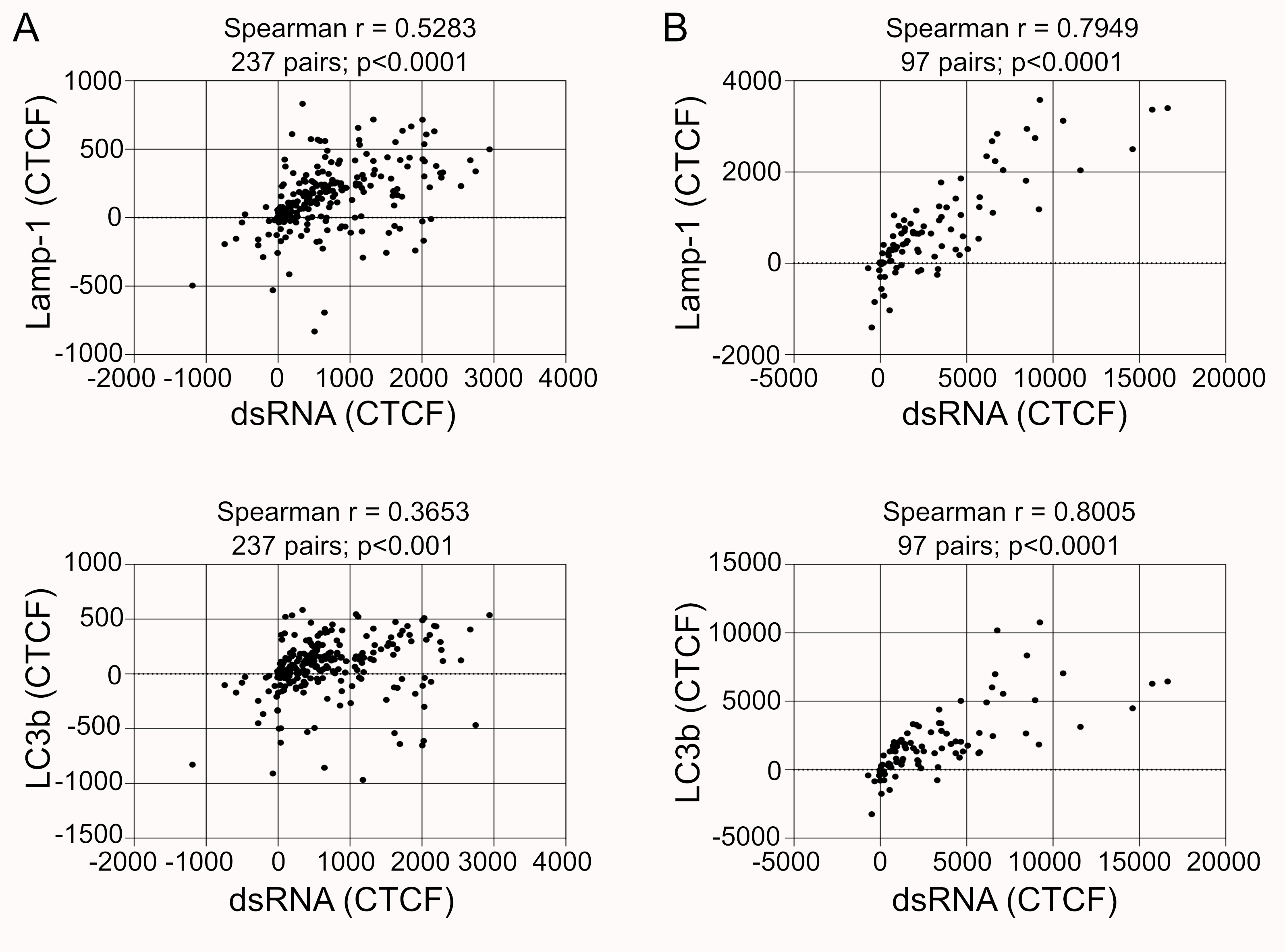

Supplement: S5 Fig — A-B: Spearman correlation analysis (two-tailed; 0.95% confidence interval) between dsRNA and Lamp-1 or LC3b fluorescence levels (CTCF) in RV-A16 (A) or RV-A2 (B) -infected HAE at 12hpi. Each dot represents the fluorescence levels of dsRNA and Lamp-1 or LC3b quantified in the same Z-slice. (TIF) [file ppat.1010159.s005.tif]

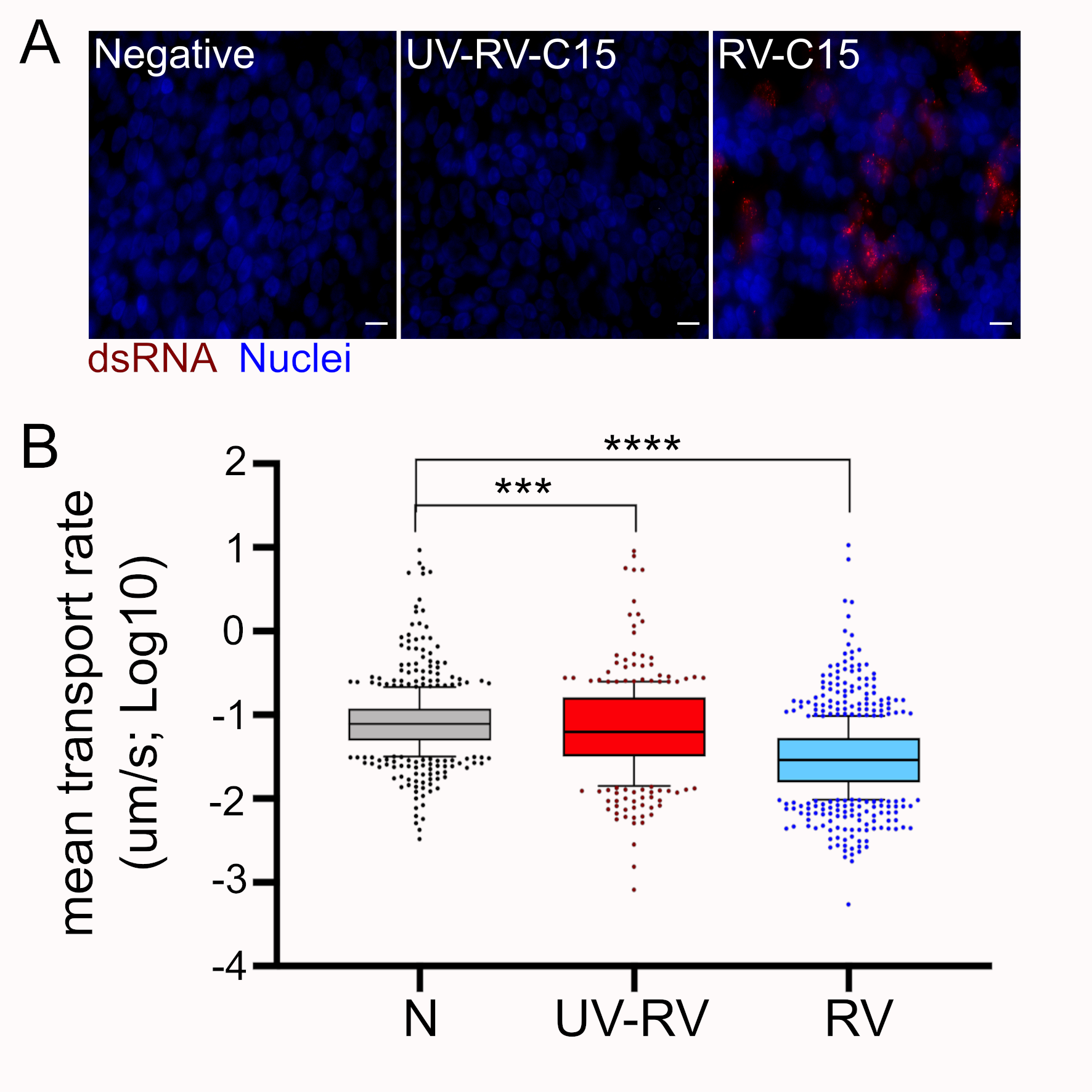

Supplement: S6 Fig — A: Immunofluorescence detection of dsRNA (red; nuclei, blue) in non-infected HAE or HAE inoculated with UV-RV-C15 or RV-C15 at 12hpi (scale bar = 10μm). B: Mean transport rate of mucus in non-infected HAE compared to HAE inoculated with UV-RV-C15 or RV-C15 at 24hpi. Box represents the 10–90 percentile values of MCC quantified in the same culture/condition at different time-points. Statistical analysis was done using Kruskal-Wallis followed by Dunn’ multi-comparison test (***p<0.001; ****p<0.0001). (TIF) [file ppat.1010159.s006.tif]
