# Supplementary material for: Rhinovirus C replication is associated with the endoplasmic reticulum and triggers cytopathic effects in an in vitro model of human airway epithelium
Source: PLoS Pathog. 2022 Jan 7;18(1):e1010159. doi: 10.1371/journal.ppat.1010159 (PMC8741012; doi:10.1371/journal.ppat.1010159)
Supplement: S1 Table — (DOCX) [file ppat.1010159.s009.docx]

**S1 Table. Pixel intensity-based and spatial (distance between center-mass) colocalization analysis between giantin and PI4P in RV-C15-infected HAE.**

| **Sample** | **PCC** | **thM1** | **thM2** | **Van Steensel's dx (pixel)** | **Giantin centroids (n)** | **PI4P centroids (n)** | **% center-mass colocalization (giantin/PI4P from total giantin)** |
| --- | --- | --- | --- | --- | --- | --- | --- |
| RV-C15 1A | 0.190 | 0.136 | 0.319 | -1 | 145 | 90 | 3.45% |
| RV-C15 1B | 0.106 | 0.071 | 0.264 | 1 | 193 | 90 | 3.63% |
| RV-C15 2A | 0.188 | 0.148 | 0.206 | 2 | 217 | 107 | 7.37% |
| RV-C15 2B | 0.251 | 0.299 | 0.265 | 2 | 56 | 95 | 8.93% |
| RV-C15 2C | 0.080 | 0.105 | 0.085 | 0 | 95 | 75 | 4.21% |
| RV-C15 3A | 0.305 | 0.226 | 0.459 | 0 | 157 | 133 | 11.46% |
| RV-C15 3B | 0.228 | 0.233 | 0.267 | -1 | 115 | 92 | 6.96% |
| RV-C15 4A | 0.187 | 0.129 | 0.367 | 2 | 275 | 69 | 2.91% |
| RV-C15 4B | 0.168 | 0.158 | 0.219 | 2 | 51 | 98 | 3.92% |
| RV-C15 4C | 0.209 | 0.212 | 0.258 | 2 | 47 | 62 | 12.77% |
| RV-C15 4D | 0.167 | 0.129 | 0.269 | 5 | 162 | 117 | 6.17% |
| RV-C15 4E | 0.023 | 0.036 | 0.025 | 1 | 25 | 67 | 4.00% |
| RV-C15 4F | 0.093 | 0.107 | 0.106 | 4 | 51 | 88 | 15.69% |
| RV-C15 5A | 0.074 | 0.066 | 0.106 | 3 | 56 | 88 | 7.14% |
| RV-C15 5B | 0.083 | 0.068 | 0.140 | -5 | 73 | 68 | 5.48% |
| RV-C15 5C | 0.092 | 0.122 | 0.082 | 1 | 42 | 60 | 9.52% |
| RV-C15 6A | 0.232 | 0.190 | 0.316 | 0 | 61 | 87 | 9.84% |
| RV-C15 6B | 0.099 | 0.131 | 0.087 | 3 | 48 | 71 | 8.33% |
| RV-C15 6C | 0.150 | 0.152 | 0.161 | -1 | 57 | 64 | 8.77% |
| **Median** | **0.167** | **0.131** | **0.219** | **1** | **61** | **88** | **7.14%** |
