# Supplementary material for: Rhinovirus C replication is associated with the endoplasmic reticulum and triggers cytopathic effects in an in vitro model of human airway epithelium
Source: PLoS Pathog. 2022 Jan 7;18(1):e1010159. doi: 10.1371/journal.ppat.1010159 (PMC8741012; doi:10.1371/journal.ppat.1010159)
Supplement: S2 Table — (DOCX) [file ppat.1010159.s010.docx]

**S2 Table. Pixel intensity-based and spatial (distance between center-mass) colocalization analysis between giantin and PI4P in RV-A16-infected HAE.**

| **Sample** | **PCC** | **thM1** | **thM2** | **Van Steensel's dx (pixel)** | **Giantin centroids (n)** | **PI4P centroids (n)** | **% center-mass colocalization (giantin/PI4P from total giantin)** |
| --- | --- | --- | --- | --- | --- | --- | --- |
| RV-A16 1A | 0.053 | 0.066 | 0.068 | -15 | 116 | 96 | 3.45% |
| RV-A16 1B | 0.133 | 0.083 | 0.266 | -4 | 40 | 83 | 2.50% |
| RV-A16 1C | 0.081 | 0.073 | 0.135 | 2 | 53 | 109 | 9.43% |
| RV-A16 2A | 0.051 | 0.076 | 0.093 | -17 | 80 | 139 | 3.75% |
| RV-A16 2B | 0.102 | 0.095 | 0.150 | 9 | 49 | 66 | 0.00% |
| RV-A16 2C | 0.062 | 0.049 | 0.109 | 1 | 45 | 46 | 2.22% |
| RV-A16 3A | 0.206 | 0.166 | 0.328 | -2 | 65 | 93 | 7.69% |
| RV-A16 3B | 0.035 | 0.028 | 0.110 | 17 | 26 | 52 | 0.00% |
| RV-A16 3C | 0.210 | 0.133 | 0.421 | 3 | 80 | 47 | 6.25% |
| RV-A16 3D | 0.192 | 0.151 | 0.271 | 1 | 36 | 49 | 0.00% |
| RV-A16 4A | 0.086 | 0.063 | 0.189 | 20 | 63 | 82 | 3.17% |
| RV-A16 4B | 0.081 | 0.046 | 0.187 | 6 | 77 | 108 | 2.60% |
| RV-A16 4C | 0.122 | 0.054 | 0.319 | -2 | 73 | 56 | 4.11% |
| **Median** | **0.086** | **0.073** | **0.187** | **1** | **63** | **82** | **3.17%** |
