# Supplementary material for: Rhinovirus C replication is associated with the endoplasmic reticulum and triggers cytopathic effects in an in vitro model of human airway epithelium
Source: PLoS Pathog. 2022 Jan 7;18(1):e1010159. doi: 10.1371/journal.ppat.1010159 (PMC8741012; doi:10.1371/journal.ppat.1010159)
Supplement: S3 Table — (DOCX) [file ppat.1010159.s011.docx]

**S3 Table. Pixel intensity-based and spatial (distance between center-mass) colocalization analysis between giantin and PI4P in RV-A2-infected HAE.**

| **Sample** | **PCC** | **thM1** | **thM2** | **Van Steensel's dx (pixel)** | **Giantin centroids (n)** | **PI4P centroids (n)** | **% center-mass colocalization (giantin/PI4P from total giantin)** |
| --- | --- | --- | --- | --- | --- | --- | --- |
| RV-A2 1A | 0.141 | 0.165 | 0.136 | 2 | 12 | 2 | 16.67% |
| RV-A2 1B | 0.116 | 0.125 | 0.122 | 4 | 50 | 3 | 6.00% |
| RV-A2 2A | 0.066 | 0.059 | 0.113 | 2 | 137 | 3 | 2.19% |
| RV-A2 2B | 0.206 | 0.216 | 0.231 | -3 | 80 | 2 | 2.50% |
| RV-A2 3A | 0.039 | 0.056 | 0.035 | 1 | 26 | 2 | 3.85% |
| RV-A2 4A | 0.028 | 0.032 | 0.041 | 5 | 81 | 3 | 3.70% |
| RV-A2 5A | 0.095 | 0.092 | 0.122 | -1 | 56 | 4 | 7.14% |
| RV-A2 6A | 0.180 | 0.279 | 0.158 | 0 | 114 | 6 | 5.26% |
| **Median** | **0.106** | **0.109** | **0.122** | **2** | **68** | **3** | **4.55%** |
