# Supplementary material for: Rhinovirus C replication is associated with the endoplasmic reticulum and triggers cytopathic effects in an in vitro model of human airway epithelium
Source: PLoS Pathog. 2022 Jan 7;18(1):e1010159. doi: 10.1371/journal.ppat.1010159 (PMC8741012; doi:10.1371/journal.ppat.1010159)
Supplement: S4 Table — (DOCX) [file ppat.1010159.s012.docx]

**S4 Table. Pixel intensity-based and spatial (distance between center-mass) colocalization analysis between dsRNA and giantin in RV-C15-infected HAE.**

| **Sample** | **PCC** | **thM1** | **thM2** | **Van Steensel's dx (pixel)** | **dsRNA centroids (n)** | **Giantin centroids (n)** | **% center-mass colocalization (dsRNA/giantin from total dsRNA)** |
| --- | --- | --- | --- | --- | --- | --- | --- |
| RV-C15 1A | 0.174 | 0.157 | 0.228 | -1 | 137 | 6 | 3.65% |
| RV-C15 1B | 0.175 | 0.153 | 0.248 | 1 | 79 | 9 | 11.39% |
| RV-C15 2A | 0.168 | 0.158 | 0.227 | 2 | 69 | 10 | 13.04% |
| RV-C15 2B | 0.237 | 0.354 | 0.191 | 2 | 93 | 11 | 11.83% |
| RV-C15 2C | 0.176 | 0.223 | 0.158 | 0 | 51 | 13 | 23.53% |
| RV-C15 3A | 0.117 | 0.120 | 0.149 | -1 | 101 | 15 | 14.85% |
| RV-C15 3B | 0.133 | 0.167 | 0.142 | 2 | 89 | 5 | 5.62% |
| RV-C15 4A | 0.094 | 0.125 | 0.102 | 1 | 83 | 7 | 8.43% |
| RV-C15 4B | 0.142 | 0.122 | 0.215 | 1 | 90 | 10 | 11.11% |
| RV-C15 4C | 0.150 | 0.165 | 0.187 | 8 | 62 | 9 | 12.90% |
| RV-C15 4D | 0.106 | 0.124 | 0.117 | 1 | 112 | 6 | 5.36% |
| RV-C15 4E | 0.105 | 0.080 | 0.168 | 0 | 99 | 2 | 2.02% |
| RV-C15 4F | 0.056 | 0.069 | 0.071 | 2 | 96 | 12 | 11.46% |
| RV-C15 5A | 0.062 | 0.044 | 0.128 | -3 | 82 | 8 | 8.54% |
| RV-C15 5B | 0.079 | 0.071 | 0.121 | -7 | 81 | 2 | 2.47% |
| RV-C15 5C | 0.102 | 0.083 | 0.162 | -1 | 104 | 5 | 4.81% |
| RV-C15 6A | 0.162 | 0.163 | 0.185 | 0 | 101 | 7 | 6.93% |
| RV-C15 6B | 0.060 | 0.056 | 0.094 | 3 | 91 | 6 | 6.59% |
| RV-C15 6C | 0.085 | 0.056 | 0.163 | 1 | 92 | 11 | 10.87% |
| **Median** | **0.117** | **0.124** | **0.162** | **1** | **91** | **8** | **8.54%** |
