# Supplementary material for: Rhinovirus C replication is associated with the endoplasmic reticulum and triggers cytopathic effects in an in vitro model of human airway epithelium
Source: PLoS Pathog. 2022 Jan 7;18(1):e1010159. doi: 10.1371/journal.ppat.1010159 (PMC8741012; doi:10.1371/journal.ppat.1010159)
Supplement: S5 Table — (DOCX) [file ppat.1010159.s013.docx]

**S5 Table. Pixel intensity-based and spatial (distance between center-mass) colocalization analysis between dsRNA and giantin in RV-A16-infected HAE.**

| **Sample** | **PCC** | **thM1** | **thM2** | **Van Steensel's dx (pixel)** | **dsRNA centroids (n)** | **Giantin centroids (n)** | **% center-mass colocalization (dsRNA/giantin from total dsRNA)** |
| --- | --- | --- | --- | --- | --- | --- | --- |
| RV-A16 1A | 0.069 | 0.056 | 0.146 | -12 | 135 | 96 | 6.67% |
| RV-A16 1B | 0.133 | 0.092 | 0.235 | 0 | 128 | 83 | 5.47% |
| RV-A16 1C | 0.069 | 0.055 | 0.147 | 2 | 101 | 109 | 3.96% |
| RV-A16 2A | 0.140 | 0.128 | 0.241 | 0 | 88 | 139 | 3.41% |
| RV-A16 2B | 0.050 | 0.049 | 0.104 | 1 | 86 | 66 | 4.65% |
| RV-A16 2C | 0.032 | 0.023 | 0.097 | 0 | 59 | 46 | 5.08% |
| RV-A16 3A | 0.161 | 0.173 | 0.194 | -14 | 49 | 93 | 10.20% |
| RV-A16 3B | 0.138 | 0.079 | 0.308 | 1 | 39 | 52 | 15.38% |
| RV-A16 3C | 0.007 | 0.024 | 0.043 | 20 | 49 | 47 | 2.04% |
| RV-A16 3D | 0.060 | 0.056 | 0.091 | -9 | 78 | 49 | 5.13% |
| RV-A16 4A | 0.105 | 0.122 | 0.116 | 4 | 108 | 82 | 6.48% |
| RV-A16 4B | 0.004 | 0.010 | 0.018 | 15 | 90 | 108 | 2.22% |
| RV-A16 4C | 0.074 | 0.045 | 0.149 | -19 | 68 | 56 | 2.94% |
| **Median** | **0.069** | **0.056** | **0.146** | **0** | **86** | **82** | **5.08%** |
