# Supplementary material for: Rhinovirus C replication is associated with the endoplasmic reticulum and triggers cytopathic effects in an in vitro model of human airway epithelium
Source: PLoS Pathog. 2022 Jan 7;18(1):e1010159. doi: 10.1371/journal.ppat.1010159 (PMC8741012; doi:10.1371/journal.ppat.1010159)
Supplement: S6 Table — (DOCX) [file ppat.1010159.s014.docx]

**S6 Table. Pixel intensity-based and spatial (distance between center-mass) colocalization analysis between dsRNA and giantin in RV-A2-infected HAE.**

| **Sample** | **PCC** | **thM1** | **thM2** | **Van Steensel's dx (pixel)** | **dsRNA centroids (n)** | **Giantin centroids (n)** | **% center-mass colocalization (dsRNA/giantin from total dsRNA)** |
| --- | --- | --- | --- | --- | --- | --- | --- |
| RV-A2 1A | 0.032 | 0.036 | 0.058 | 2 | 113 | 4 | 3.54% |
| RV-A2 1B | 0.045 | 0.038 | 0.088 | 14 | 94 | 4 | 4.26% |
| RV-A2 2A | 0.053 | 0.058 | 0.077 | 1 | 78 | 9 | 11.54% |
| RV-A2 2B | 0.046 | 0.074 | 0.054 | -19 | 97 | 5 | 5.15% |
| RV-A2 3A | 0.044 | 0.029 | 0.108 | 1 | 77 | 1 | 1.30% |
| RV-A2 4A | 0.087 | 0.066 | 0.140 | 0 | 116 | 10 | 8.62% |
| RV-A2 5A | 0.075 | 0.046 | 0.190 | 19 | 160 | 12 | 7.50% |
| RV-A2 6A | 0.106 | 0.179 | 0.110 | 10 | 96 | 5 | 5.21% |
| **Median** | **0.050** | **0.052** | **0.098** | **2** | **97** | **5** | **5.18%** |
