# Supplementary material for: Rhinovirus C replication is associated with the endoplasmic reticulum and triggers cytopathic effects in an in vitro model of human airway epithelium
Source: PLoS Pathog. 2022 Jan 7;18(1):e1010159. doi: 10.1371/journal.ppat.1010159 (PMC8741012; doi:10.1371/journal.ppat.1010159)
Supplement: S7 Table — (DOCX) [file ppat.1010159.s015.docx]

**S7 Table. Pixel intensity-based and spatial (distance between center-mass) colocalization analysis between dsRNA and PI4P in RV-C15-infected HAE.**

| **Sample** | **PCC** | **thM1** | **thM2** | **Van Steensel's dx (pixel)** | **dsRNA centroids (n)** | **PI4P centroids (n)** | **% center-mass colocalization (dsRNA/PI4P from total dsRNA)** |
| --- | --- | --- | --- | --- | --- | --- | --- |
| RV-C15 1A | 0.259 | 0.357 | 0.220 | 0 | 137 | 6 | 4.38% |
| RV-C15 1B | 0.108 | 0.224 | 0.098 | -2 | 79 | 3 | 3.80% |
| RV-C15 2A | 0.281 | 0.371 | 0.256 | 0 | 69 | 6 | 10.14% |
| RV-C15 2B | 0.280 | 0.385 | 0.235 | -1 | 93 | 4 | 4.30% |
| RV-C15 2C | 0.051 | 0.068 | 0.060 | -1 | 51 | 5 | 9.80% |
| RV-C15 3A | 0.212 | 0.298 | 0.182 | 0 | 101 | 8 | 7.92% |
| RV-C15 3B | 0.230 | 0.290 | 0.215 | -1 | 89 | 4 | 4.49% |
| RV-C15 4A | 0.253 | 0.519 | 0.148 | 0 | 83 | 18 | 19.28% |
| RV-C15 4B | 0.227 | 0.220 | 0.280 | -1 | 90 | 10 | 11.11% |
| RV-C15 4C | 0.292 | 0.327 | 0.305 | -1 | 62 | 12 | 19.35% |
| RV-C15 4D | 0.244 | 0.388 | 0.176 | 0 | 118 | 3 | 2.54% |
| RV-C15 4E | 0.040 | 0.029 | 0.087 | -2 | 99 | 2 | 2.02% |
| RV-C15 4F | 0.052 | 0.065 | 0.067 | 0 | 96 | 3 | 3.13% |
| RV-C15 5A | 0.211 | 0.168 | 0.302 | -2 | 82 | 7 | 7.32% |
| RV-C15 5B | 0.092 | 0.122 | 0.101 | -1 | 81 | 2 | 2.47% |
| RV-C15 5C | 0.082 | 0.055 | 0.161 | -1 | 104 | 4 | 3.85% |
| RV-C15 6A | 0.295 | 0.371 | 0.254 | 0 | 101 | 8 | 7.92% |
| RV-C15 6B | 0.144 | 0.097 | 0.243 | 0 | 91 | 8 | 8.79% |
| RV-C15 6C | 0.130 | 0.085 | 0.233 | 6 | 92 | 12 | 10.87% |
| **Median** | **0.212** | **0.224** | **0.215** | **-1** | **91** | **6** | **7.32%** |
