# Supplementary material for: Rhinovirus C replication is associated with the endoplasmic reticulum and triggers cytopathic effects in an in vitro model of human airway epithelium
Source: PLoS Pathog. 2022 Jan 7;18(1):e1010159. doi: 10.1371/journal.ppat.1010159 (PMC8741012; doi:10.1371/journal.ppat.1010159)
Supplement: S8 Table — (DOCX) [file ppat.1010159.s016.docx]

**S8 Table. Pixel intensity-based and spatial (distance between center-mass) colocalization analysis between dsRNA and PI4P in RV-A16-infected HAE.**

| **Sample** | **PCC** | **thM1** | **thM2** | **Van Steensel's dx (pixel)** | **dsRNA centroids (n)** | **PI4P centroids (n)** | **% center-mass colocalization (dsRNA/PI4P from total dsRNA)** |
| --- | --- | --- | --- | --- | --- | --- | --- |
| RV-A16 1A | 0.068 | 0.056 | 0.142 | -1 | 135 | 116 | 6.67% |
| RV-A16 1B | 0.105 | 0.146 | 0.117 | 1 | 126 | 40 | 3.97% |
| RV-A16 1C | 0.194 | 0.184 | 0.262 | -1 | 101 | 53 | 2.97% |
| RV-A16 2A | 0.095 | 0.111 | 0.170 | -1 | 88 | 80 | 0.00% |
| RV-A16 2B | 0.099 | 0.108 | 0.144 | 0 | 86 | 49 | 4.65% |
| RV-A16 2C | 0.048 | 0.052 | 0.098 | 0 | 59 | 45 | 3.39% |
| RV-A16 3A | 0.243 | 0.359 | 0.204 | 2 | 49 | 65 | 6.12% |
| RV-A16 3B | 0.116 | 0.154 | 0.154 | -13 | 39 | 26 | 7.69% |
| RV-A16 3C | 0.184 | 0.296 | 0.165 | 0 | 49 | 80 | 2.04% |
| RV-A16 3D | 0.193 | 0.218 | 0.195 | -1 | 78 | 36 | 6.41% |
| RV-A16 4A | 0.196 | 0.382 | 0.122 | 0 | 108 | 63 | 2.78% |
| RV-A16 4B | 0.112 | 0.192 | 0.084 | 0 | 90 | 77 | 2.22% |
| RV-A16 4C | 0.201 | 0.290 | 0.163 | 2 | 68 | 73 | 5.88% |
| **Median** | **0.116** | **0.184** | **0.154** | **0** | **86** | **63** | **3.97%** |
