# Supplementary material for: Rhinovirus C replication is associated with the endoplasmic reticulum and triggers cytopathic effects in an in vitro model of human airway epithelium
Source: PLoS Pathog. 2022 Jan 7;18(1):e1010159. doi: 10.1371/journal.ppat.1010159 (PMC8741012; doi:10.1371/journal.ppat.1010159)
Supplement: S9 Table — (DOCX) [file ppat.1010159.s017.docx]

**S9 Table. Pixel intensity-based and spatial (distance between center-mass) colocalization analysis between dsRNA and PI4P in RV-A2-infected HAE.**

| **Sample** | **PCC** | **thM1** | **thM2** | **Van Steensel's dx (pixel)** | **dsRNA centroids (n)** | **PI4P centroids (n)** | **% center-mass colocalization (dsRNA/PI4P from total dsRNA)** |
| --- | --- | --- | --- | --- | --- | --- | --- |
| RV-A2 1A | 0.072 | 0.060 | 0.118 | -1 | 113 | 12 | 1.77% |
| RV-A2 1B | 0.077 | 0.058 | 0.137 | -2 | 94 | 50 | 3.19% |
| RV-A2 2A | 0.110 | 0.152 | 0.106 | -1 | 78 | 137 | 12.82% |
| RV-A2 2B | 0.118 | 0.163 | 0.111 | -1 | 97 | 80 | 6.19% |
| RV-A2 3A | 0.049 | 0.024 | 0.141 | -1 | 77 | 26 | 2.60% |
| RV-A2 4A | 0.079 | 0.069 | 0.115 | -2 | 116 | 81 | 4.31% |
| RV-A2 5A | 0.081 | 0.058 | 0.182 | 0 | 160 | 56 | 5.63% |
| RV-A2 6A | 0.211 | 0.224 | 0.243 | -1 | 93 | 114 | 16.13% |
| **Median** | **0.080** | **0.065** | **0.128** | **-1** | **96** | **68** | **4.97%** |
