# Supplementary material for: Rhinovirus C replication is associated with the endoplasmic reticulum and triggers cytopathic effects in an in vitro model of human airway epithelium
Source: PLoS Pathog. 2022 Jan 7;18(1):e1010159. doi: 10.1371/journal.ppat.1010159 (PMC8741012; doi:10.1371/journal.ppat.1010159)
Supplement: S10 Table — (DOCX) [file ppat.1010159.s018.docx]

**S10 Table. Pixel intensity-based and spatial (distance between center-mass) colocalization analysis between dsRNA and calnexin in RV-C15-infected HAE.**

| **Sample** | **PCC** | **thM1** | **thM2** | **Van Steensel's dx (pixel)** | **dsRNA centroids (n)** | **Calnexin centroids (n)** | **% center-mass colocalization (dsRNA/calnexin from total dsRNA)** |
| --- | --- | --- | --- | --- | --- | --- | --- |
| RV-C15 1A | 0.53 | 0.63 | 0.47 | 2 | 142 | 188 | 54.93% |
| RV-C15 1B | 0.49 | 0.51 | 0.49 | 2 | 120 | 150 | 41.67% |
| RV-C15 2C | 0.19 | 0.13 | 0.34 | 1 | 1233 | 208 | 4.38% |
| RV-C15 2D | 0.41 | 0.6 | 0.29 | 2 | 73 | 184 | 28.77% |
| RV-C15 3E | 0.29 | 0.55 | 0.16 | 1 | 122 | 271 | 45.08% |
| RV-C15 4F | 0.35 | 0.57 | 0.22 | 1 | 112 | 212 | 55.36% |
| **Median** | **0.381** | **0.563** | **0.317** | **1.5** | **121** | **198** | **43.37%** |
