# Supplementary material for: Rhinovirus C replication is associated with the endoplasmic reticulum and triggers cytopathic effects in an in vitro model of human airway epithelium
Source: PLoS Pathog. 2022 Jan 7;18(1):e1010159. doi: 10.1371/journal.ppat.1010159 (PMC8741012; doi:10.1371/journal.ppat.1010159)
Supplement: S11 Table — (DOCX) [file ppat.1010159.s019.docx]

**S11 Table. Pixel intensity-based and spatial (distance between center-mass) colocalization analysis between dsRNA and calnexin in RV-A16-infected HAE.**

| **Sample** | **PCC** | **thM1** | **thM2** | **Van Steensel's dx (pixel)** | **dsRNA centroids (n)** | **Calnexin centroids (n)** | **% center-mass colocalization (dsRNA/calnexin from total dsRNA)** |
| --- | --- | --- | --- | --- | --- | --- | --- |
| RV-A16 1A | 0.366 | 0.407 | 0.387 | 1 | 141 | 385 | 14.18% |
| RV-A16 1B | 0.289 | 0.372 | 0.292 | 3 | 128 | 274 | 10.16% |
| RV-A16 1C | 0.265 | 0.328 | 0.271 | 2 | 101 | 161 | 2.97% |
| RV-A16 1D | 0.340 | 0.231 | 0.607 | 3 | 410 | 289 | 2.68% |
| RV-A16 1E | 0.287 | 0.276 | 0.374 | 2 | 184 | 365 | 3.26% |
| RV-A16 2F | 0.352 | 0.263 | 0.618 | 2 | 286 | 217 | 3.15% |
| RV-A16 2G | 0.320 | 0.236 | 0.584 | 4 | 241 | 180 | 0.83% |
| RV-A16 2H | 0.358 | 0.255 | 0.637 | 3 | 362 | 235 | 0.55% |
| RV-A16 3I | 0.339 | 0.389 | 0.359 | 2 | 85 | 142 | 22.35% |
| RV-A16 3J | 0.272 | 0.315 | 0.312 | 2 | 285 | 139 | 1.05% |
| RV-A16 3K | 0.364 | 0.280 | 0.633 | 0 | 99 | 185 | 4.04% |
| **Median** | **0.339** | **0.280** | **0.387** | **2** | **184** | **217** | **3.15%** |
