# Supplementary material for: Rhinovirus C replication is associated with the endoplasmic reticulum and triggers cytopathic effects in an in vitro model of human airway epithelium
Source: PLoS Pathog. 2022 Jan 7;18(1):e1010159. doi: 10.1371/journal.ppat.1010159 (PMC8741012; doi:10.1371/journal.ppat.1010159)
Supplement: S12 Table — (DOCX) [file ppat.1010159.s020.docx]

**S12 Table. Pixel intensity-based and spatial (distance between center-mass) colocalization analysis between dsRNA and calnexin in RV-A2-infected HAE.**

| **Sample** | **PCC** | **thM1** | **thM2** | **Van Steensel's dx (pixel)** | **dsRNA centroids (n)** | **Calnexin centroids (n)** | **% center-mass colocalization (dsRNA/calnexin from total dsRNA)** |
| --- | --- | --- | --- | --- | --- | --- | --- |
| RV-A2 1A | 0.3 | 0.5 | 0.22 | 2 | 224 | 292 | 1.79% |
| RV-A2 2B | 0.37 | 0.58 | 0.29 | -1 | 95 | 175 | 8.42% |
| RV-A2 3C | 0.43 | 0.51 | 0.44 | 2 | 128 | 82 | 1.56% |
| RV-A2 4D | 0.45 | 0.48 | 0.52 | 2 | 173 | 32 | 1.73% |
| RV-A2 6E | 0.33 | 0.47 | 0.29 | 2 | 246 | 74 | 2.44% |
| RV-A2 7A | 0.28 | 0.48 | 0.22 | 1 | 155 | 108 | 2.58% |
| **Median** | **0.353** | **0.491** | **0.288** | **2** | **164** | **95** | **2.11%** |
