# Supplementary material for: Rhinovirus C replication is associated with the endoplasmic reticulum and triggers cytopathic effects in an in vitro model of human airway epithelium
Source: PLoS Pathog. 2022 Jan 7;18(1):e1010159. doi: 10.1371/journal.ppat.1010159 (PMC8741012; doi:10.1371/journal.ppat.1010159)
Supplement: S13 Table — (DOCX) [file ppat.1010159.s021.docx]

**S13 Table. Pixel intensity-based and spatial (distance between center-mass) colocalization analysis between dsRNA and giantin in RV-C15-infected HAE.**

| **Sample** | **PCC** | **thM1** | **thM2** | **Van Steensel's dx (pixel)** | **dsRNA centroids (n)** | **Giantin centroids (n)** | **% center-mass colocalization (dsRNA/giantin from total dsRNA)** |
| --- | --- | --- | --- | --- | --- | --- | --- |
| RV-C15 1A | 0.14 | 0.15 | 0.15 | -1 | 142 | 269 | 18.31% |
| RV-C15 1B | 0.19 | 0.2 | 0.2 | -3 | 120 | 221 | 12.50% |
| RV-C15 2C | 0.07 | 0.06 | 0.15 | 1 | 1233 | 326 | 2.51% |
| RV-C15 2D | 0.13 | 0.2 | 0.1 | 0 | 73 | 270 | 10.96% |
| RV-C15 3E | 0.09 | 0.17 | 0.06 | -2 | 122 | 281 | 24.59% |
| RV-C15 4F | 0.15 | 0.23 | 0.11 | -1 | 111 | 161 | 25.23% |
| **Median** | **0.136** | **0.183** | **0.130** | **-1** | **121** | **269.5** | **15.40%** |
