# Supplementary material for: Rhinovirus C replication is associated with the endoplasmic reticulum and triggers cytopathic effects in an in vitro model of human airway epithelium
Source: PLoS Pathog. 2022 Jan 7;18(1):e1010159. doi: 10.1371/journal.ppat.1010159 (PMC8741012; doi:10.1371/journal.ppat.1010159)
Supplement: S14 Table — (DOCX) [file ppat.1010159.s022.docx]

**S14 Table. Pixel intensity-based and spatial (distance between center-mass) colocalization analysis between dsRNA and giantin in RV-A16-infected HAE.**

| **Sample** | **PCC** | **thM1** | **thM2** | **Van Steensel's dx (pixel)** | **dsRNA centroids (n)** | **Giantin centroids (n)** | **% center-mass colocalization (dsRNA/giantin from total dsRNA)** |
| --- | --- | --- | --- | --- | --- | --- | --- |
| RV-A16 1A | 0.372 | 0.501 | 0.333 | -3 | 141 | 14 | 10.64% |
| RV-A16 1B | 0.331 | 0.441 | 0.313 | 2 | 128 | 2 | 1.56% |
| RV-A16 1C | 0.363 | 0.607 | 0.264 | -1 | 101 | 7 | 6.93% |
| RV-A16 1D | 0.241 | 0.306 | 0.311 | 1 | 410 | 1 | 0.24% |
| RV-A16 1E | 0.269 | 0.480 | 0.223 | -1 | 184 | 26 | 14.67% |
| RV-A16 2F | 0.319 | 0.346 | 0.450 | -1 | 286 | 5 | 2.45% |
| RV-A16 2G | 0.376 | 0.378 | 0.515 | 5 | 241 | 8 | 3.32% |
| RV-A16 2H | 0.241 | 0.318 | 0.337 | 0 | 362 | 0 | 0.00% |
| RV-A16 3I | 0.115 | 0.172 | 0.153 | 0 | 85 | 7 | 8.24% |
| RV-A16 3J | 0.215 | 0.311 | 0.228 | 5 | 285 | 69 | 21.05% |
| RV-A16 3K | 0.259 | 0.260 | 0.440 | -18 | 99 | 1 | 1.01% |
| **Median** | **0.269** | **0.346** | **0.313** | **0** | **184** | **7** | **3.32%** |
