# Supplementary material for: Rhinovirus C replication is associated with the endoplasmic reticulum and triggers cytopathic effects in an in vitro model of human airway epithelium
Source: PLoS Pathog. 2022 Jan 7;18(1):e1010159. doi: 10.1371/journal.ppat.1010159 (PMC8741012; doi:10.1371/journal.ppat.1010159)
Supplement: S15 Table — (DOCX) [file ppat.1010159.s023.docx]

**S15 Table. Pixel intensity-based and spatial (distance between center-mass) colocalization analysis between dsRNA and giantin in RV-A2-infected HAE.**

| **Sample** | **PCC** | **thM1** | **thM2** | **Van Steensel's dx (pixel)** | **dsRNA centroids (n)** | **Giantin centroids (n)** | **% center-mass colocalization (dsRNA/giantin from total dsRNA)** |
| --- | --- | --- | --- | --- | --- | --- | --- |
| RV-A2 1A | 0.19 | 0.27 | 0.21 | 0 | 608 | 628 | 4.11% |
| RV-A2 2B | 0.33 | 0.52 | 0.27 | -3 | 95 | 120 | 2.11% |
| RV-A2 3C | 0.27 | 0.3 | 0.33 | 2 | 128 | 253 | 1.56% |
| RV-A2 4D | 0.34 | 0.36 | 0.44 | 0 | 173 | 104 | 8.67% |
| RV-A2 6E | 0.26 | 0.39 | 0.23 | -1 | 246 | 140 | 7.32% |
| RV-A2 7A | 0.22 | 0.39 | 0.18 | 1 | 155 | 132 | 2.58% |
| **Median** | **0.266** | **0.378** | **0.249** | **0** | **164** | **136** | **3.35%** |
