# Supplementary material for: Rhinovirus C replication is associated with the endoplasmic reticulum and triggers cytopathic effects in an in vitro model of human airway epithelium
Source: PLoS Pathog. 2022 Jan 7;18(1):e1010159. doi: 10.1371/journal.ppat.1010159 (PMC8741012; doi:10.1371/journal.ppat.1010159)
Supplement: S16 Table — (DOCX) [file ppat.1010159.s024.docx]

**S16 Table. Pixel intensity-based and spatial (distance between center-mass) colocalization analysis between dsRNA and STING in RV-C15-infected HAE.**

| **Sample** | **PCC** | **thM1** | **thM2** | **Van Steensel's dx (pixel)** | **dsRNA centroids (n)** | **STING centroids (n)** | **% center-mass colocalization (dsRNA/STING from total dsRNA)** |
| --- | --- | --- | --- | --- | --- | --- | --- |
| RV-C15 1A | 0.241 | 0.256 | 0.288 | 0 | 107 | 91 | 3.74% |
| RV-C15 1B | 0.363 | 0.492 | 0.326 | 2 | 109 | 54 | 0.92% |
| RV-C15 1C | 0.297 | 0.207 | 0.597 | -4 | 80 | 74 | 11.25% |
| RV-C15 1D | 0.025 | 0.029 | 0.054 | 2 | 150 | 77 | 6.00% |
| RV-C15 1E | 0.055 | 0.055 | 0.090 | 3 | 136 | 92 | 3.68% |
| RV-C15 1F | 0.041 | 0.044 | 0.087 | 0 | 101 | 55 | 2.97% |
| RV-C15 1G | 0.060 | 0.030 | 0.163 | -2 | 151 | 59 | 1.32% |
| RV-C15 1H | 0.005 | 0.023 | 0.021 | -1 | 137 | 98 | 1.46% |
| RV-C15 2A | 0.321 | 0.406 | 0.319 | -1 | 53 | 46 | 7.55% |
| RV-C15 2B | 0.367 | 0.395 | 0.455 | -1 | 61 | 37 | 6.56% |
| RV-C15 2C | 0.239 | 0.197 | 0.333 | 2 | 138 | 51 | 2.90% |
| RV-C15 2D | 0.212 | 0.232 | 0.300 | -7 | 54 | 80 | 3.70% |
| RV-C15 2E | 0.005 | 0.021 | 0.049 | -3 | 116 | 78 | 5.17% |
| RV-C15 2F | 0.127 | 0.096 | 0.213 | -1 | 86 | 103 | 4.65% |
| RV-C15 3A | 0.402 | 0.471 | 0.400 | 2 | 42 | 33 | 4.76% |
| RV-C15 3B | 0.314 | 0.268 | 0.466 | -3 | 49 | 62 | 2.04% |
| RV-C15 3C | 0.265 | 0.266 | 0.325 | 0 | 61 | 36 | 3.28% |
| RV-C15 3D | 0.198 | 0.152 | 0.300 | 1 | 81 | 55 | 4.94% |
| RV-C15 3E | 0.455 | 0.631 | 0.371 | 1 | 51 | 16 | 0.00% |
| RV-C15 3F | 0.149 | 0.292 | 0.101 | 1 | 70 | 52 | 4.29% |
| **Median** | **0.226** | **0.220** | **0.300** | **0** | **83.5** | **57** | **3.72%** |
