# Supplementary material for: Rhinovirus C replication is associated with the endoplasmic reticulum and triggers cytopathic effects in an in vitro model of human airway epithelium
Source: PLoS Pathog. 2022 Jan 7;18(1):e1010159. doi: 10.1371/journal.ppat.1010159 (PMC8741012; doi:10.1371/journal.ppat.1010159)
Supplement: S17 Table — (DOCX) [file ppat.1010159.s025.docx]

**S17 Table. Pixel intensity-based and spatial (distance between center-mass) colocalization analysis between Lamp-1 and LC3b in RV-C15-infected HAE.**

| **Sample** | **PCC** | **thM1** | **thM2** | **Van Steensel's dx (pixel)** | **Lamp-1 centroids (n)** | **LC3b centroids (n)** | **% center-mass colocalization (Lamp-1/LC3b from total Lamp-1)** |
| --- | --- | --- | --- | --- | --- | --- | --- |
| RV-C15 1A | 0.262 | 0.129 | 0.570 | -1 | 58 | 49 | 5.17% |
| RV-C15 1B | 0.040 | 0.032 | 0.098 | -1 | 70 | 98 | 1.43% |
| RV-C15 1C | 0.159 | 0.114 | 0.300 | 0 | 56 | 134 | 3.57% |
| RV-C15 1D | 0.048 | 0.016 | 0.240 | 0 | 9 | 20 | 0.00% |
| RV-C15 2A | 0.058 | 0.034 | 0.183 | -2 | 22 | 154 | 4.55% |
| RV-C15 2B | 0.256 | 0.120 | 0.631 | -1 | 18 | 48 | 16.67% |
| RV-C15 2C | 0.027 | 0.016 | 0.107 | -1 | 50 | 44 | 0.00% |
| RV-C15 3A | 0.148 | 0.037 | 0.684 | -1 | 24 | 27 | 0.00% |
| RV-C15 3B | 0.157 | 0.047 | 0.620 | -1 | 98 | 33 | 3.06% |
| RV-C15 3C | 0.107 | 0.023 | 0.596 | -2 | 40 | 27 | 12.50% |
| RV-C15 3D | 0.186 | 0.071 | 0.577 | -2 | 32 | 35 | 3.13% |
| RV-C15 4A | 0.128 | 0.030 | 0.677 | -2 | 40 | 37 | 2.50% |
| RV-C15 4B | 0.142 | 0.040 | 0.597 | -2 | 29 | 40 | 0.00% |
| RV-C15 4C | 0.186 | 0.061 | 0.690 | -3 | 40 | 41 | 2.50% |
| RV-C15 5A | 0.249 | 0.082 | 0.792 | -1 | 77 | 26 | 2.60% |
| RV-C15 5B | 0.067 | 0.010 | 0.534 | -1 | 45 | 7 | 0.00% |
| RV-C15 6A | 0.321 | 0.153 | 0.723 | -1 | 32 | 30 | 3.13% |
| RV-C15 6B | 0.054 | 0.012 | 0.323 | 0 | 23 | 10 | 0.00% |
| RV-C15 6C | 0.024 | 0.007 | 0.129 | -1 | 62 | 0 | 0.00% |
| **Median** | **0.142** | **0.037** | **0.577** | **-1** | **40** | **35** | **2.50%** |
