# Supplementary material for: Rhinovirus C replication is associated with the endoplasmic reticulum and triggers cytopathic effects in an in vitro model of human airway epithelium
Source: PLoS Pathog. 2022 Jan 7;18(1):e1010159. doi: 10.1371/journal.ppat.1010159 (PMC8741012; doi:10.1371/journal.ppat.1010159)
Supplement: S18 Table — (DOCX) [file ppat.1010159.s026.docx]

**S18 Table. Pixel intensity-based and spatial (distance between center-mass) colocalization analysis between Lamp-1 and LC3b in RV-A16-infected HAE.**

| **Sample** | **PCC** | **thM1** | **thM2** | **Van Steensel's dx (pixel)** | **Lamp-1 centroids (n)** | **LC3b centroids (n)** | **% center-mass colocalization (Lamp-1/LC3b from total Lamp-1)** |
| --- | --- | --- | --- | --- | --- | --- | --- |
| RV-A16 1A | 0.604 | 0.670 | 0.569 | -1 | 42 | 60 | 16.67% |
| RV-A16 1B | 0.388 | 0.585 | 0.296 | -1 | 32 | 124 | 9.38% |
| RV-A16 1C | 0.399 | 0.635 | 0.290 | -2 | 18 | 92 | 0.00% |
| RV-A16 3A | 0.510 | 0.610 | 0.488 | -1 | 29 | 46 | 24.14% |
| RV-A16 3B | 0.435 | 0.584 | 0.356 | -1 | 41 | 116 | 17.07% |
| RV-A16 3C | 0.409 | 0.557 | 0.338 | -2 | 48 | 55 | 8.33% |
| RV-A16 3D | 0.583 | 0.665 | 0.538 | -2 | 22 | 44 | 27.27% |
| RV-A16 4A | 0.568 | 0.647 | 0.530 | -1 | 60 | 49 | 16.67% |
| RV-A16 4C | 0.535 | 0.618 | 0.493 | -1 | 27 | 32 | 7.41% |
| RV-A16 5A | 0.395 | 0.590 | 0.308 | -2 | 51 | 160 | 13.73% |
| RV-A16 5B | 0.418 | 0.605 | 0.318 | -2 | 35 | 40 | 2.86% |
| RV-A16 5C | 0.392 | 0.599 | 0.304 | -2 | 35 | 96 | 17.14% |
| RV-A16 5D | 0.456 | 0.639 | 0.376 | -2 | 41 | 103 | 7.32% |
| **Median** | **0.435** | **0.610** | **0.356** | **-2** | **35** | **60** | **13.73%** |
