# Supplementary material for: Rhinovirus C replication is associated with the endoplasmic reticulum and triggers cytopathic effects in an in vitro model of human airway epithelium
Source: PLoS Pathog. 2022 Jan 7;18(1):e1010159. doi: 10.1371/journal.ppat.1010159 (PMC8741012; doi:10.1371/journal.ppat.1010159)
Supplement: S19 Table — (DOCX) [file ppat.1010159.s027.docx]

**S19 Table. Pixel intensity-based and spatial (distance between center-mass) colocalization analysis between Lamp-1 and LC3b in RV-A2-infected HAE.**

| **Sample** | **PCC** | **thM1** | **thM2** | **Van Steensel's dx (pixel)** | **Lamp-1 centroids (n)** | **LC3b centroids (n)** | **% center-mass colocalization (Lamp-1/LC3b from total Lamp-1)** |
| --- | --- | --- | --- | --- | --- | --- | --- |
| RV-A2 1A | 0.361 | 0.372 | 0.388 | 0 | 123 | 56 | 9.76% |
| RV-A2 2A | 0.370 | 0.175 | 0.869 | -1 | 47 | 50 | 2.13% |
| RV-A2 3A | 0.393 | 0.281 | 0.601 | -1 | 79 | 98 | 3.80% |
| RV-A2 3B | 0.228 | 0.225 | 0.299 | 0 | 64 | 41 | 0.00% |
| RV-A2 4A | 0.166 | 0.096 | 0.367 | -2 | 31 | 84 | 0.00% |
| RV-A2 4b | 0.141 | 0.119 | 0.231 | -2 | 18 | 55 | 0.00% |
| RV-A2 4C | 0.135 | 0.120 | 0.242 | -3 | 49 | 67 | 4.08% |
| **Median** | **0.228** | **0.175** | **0.367** | **-1** | **49** | **56** | **2.13%** |
